# The Impact of Vitamin D in the Treatment of Essential Hypertension

**DOI:** 10.3390/ijms19020455

**Published:** 2018-02-03

**Authors:** Christian Legarth, Daniela Grimm, Markus Wehland, Johann Bauer, Marcus Krüger

**Affiliations:** 1 Institute of Biomedicine, Pharmacology, Aarhus University, Wilhelm Meyers Allé 4, DK-8000 Aarhus C, Denmark; chr_brod@hotmail.com; 2 Clinic for Plastic, Aesthetic and Hand Surgery, Otto von Guericke University, Leipziger Str. 44, 39120 Magdeburg, Germany; markus.wehland@med.ovgu.de (M.W.); marcus.krueger@med.ovgu.de (M.K.); 3 Max Planck Institute for Biochemistry, Am Klopferspitz 18, 82152 Martinsried, Germany; jbauer@biochem.mpg.de

**Keywords:** vitamin D, hypertension, essential hypertension, renin-angiotensin-aldosterone system (RAAS), cholecalciferol

## Abstract

The aim of this review is to investigate, whether there is a possible link between vitamin D supplementation and the reduction of blood pressure in hypertensive patients. The renin-angiotensin-aldosterone system is known for being deeply involved in cardiovascular tonus and blood pressure regulation. Hence, many of the pharmaceutical antihypertensive drugs inhibit this system. Interestingly, experimental studies in mice have indicated that vitamin D supplementation significantly lowers renin synthesis and blood pressure. It is conceivable that similar mechanisms may be found in the human organism. Regarding this, large-scale cross-sectional studies suggest the serum 25(OH)D-level to be inversely correlated to the prevalence of hypertension. However, randomized controlled trials (RCTs) have not found a clear association between vitamin D supplementation and improvements in hypertension. Nevertheless, the missing association of vitamin D and hypertension in clinical trials can be due to suboptimal study designs. There are hints that restoration of serum 25(OH)D levels during vitamin D therapy is essential to achieve possible beneficial cardiovascular effects. It is important to perform long-term trials with a short dose interval and a high bioavailability of supplementation. Taken together, more RCTs are required to further investigate if vitamin D can be beneficial for the reduction of blood pressure.

## 1. Introduction

According to the World Health Organization (WHO), one in three adults worldwide has raised blood pressure—a condition that causes around half of all deaths from stroke and heart disease [1]. Adiposity, lack of physical activity and excessive salt intake are some of the best-known environmental factors associated with hypertension. In recent years, yet another cause has been postulated: vitamin D deficiency [2,3,4,5,6]. Vitamin D is a key player in calcium homeostasis, in maintaining optimal bone metabolism and reducing fracture risk [7]. Several studies indicate that vitamin D also seems to play a protective role against the development of hypertension [5,8]. In this review, we summarize the existing literature that is concerned with vitamin D and hypertension and investigate if vitamin D (supplements) could be a beneficial treatment agent for hypertensive individuals.

For the literature search, the online databases PubMed (http://www.ncbi.nlm.nih.gov/pubmed/), Scopus (http://www.scopus.com/) and clinicaltrials.gov (http://clinicaltrials.gov/) were used up to January 2018. Search terms such as; “(Cholecalciferol OR vitamin D) and hypertension”; “Essential hypertension and (cholecalciferol OR ergocalciferol)”; “Vitamin D deficiency and hypertension”, have been applied in the online databases. There were no restrictions in language set up. In PubMed, a search for “Essential hypertension” gave 30986 items, “Vitamin D” 74983 items and “hypertension and vitamin D” gave 1950 items accessed on 29 January 2018.

## 2. Arterial Hypertension

### 2.1. Definition, Causes and Risks

Arterial hypertension belongs to the most prevalent diseases and accounts for about 7.5 million deaths per year (about 13% of all deaths) worldwide. According to the WHO, hypertension is a major risk factor for the development of a variety of diseases, including cardiovascular diseases (CVD), kidney failure, cognitive impairment etc. [1]. It is estimated that approximately 22% (about 1 billion individuals) of the adult population above 25 years of age worldwide suffer from arterial hypertension [1]. In Europe, hypertension affects about 30–45% of people as reported in 2013 [9]; a large proportion is still unaware of their condition and left untreated [10].

Hypertension is a condition, in which either the systolic blood pressure (SBP) and/or diastolic blood pressure (DBP) stays elevated persistently. An exact definition has been difficult to establish. However, in 2017 the American College of Cardiology has newly defined hypertension in the Guidelines for the Prevention, Detection, Evaluation, and Management of High Blood Pressure in Adults [11]. The new classification in given in Table 1.

Approximately 95% of all cases of arterial hypertension can be classified as essential hypertension (EH). This condition is characterized by an imbalance between vascular tonus and cardiac output without any identifiable cause.

The mean arterial BP (MAP) can be derived analogously from Ohm’s law [12], which states:

MAP = Cardiac Output (CO) × Total Peripheral Resistance (TPR)(1)

An increase in MAP can lead to small artery remodelling, which increases the media-to-lumen ratio, TPR, causes wall damage and reduces distensibility in large arteries. These alterations finally lead to vessel wall hypertrophy and further elevate SBP—a vicious circle is created [13].

Age, smoking, high Body Mass Index (BMI), a salty diet and genetic dispositions are some of the known environmental and hereditary precursors linked to EH. Hence, it is important to eliminate the triggers, increase level of physical exercise and normalize BMI. The importance of preventing hypertension is underpinned by the findings of Lewington et al. [14]. They found that a 2 mmHg lowering of normal SBP could give an approximately 10% reduced risk of stroke mortality [14].

### 2.2. Management of Hypertension

In addition to lifestyle changes, pharmaceutical treatment may be relevant. Thiazide diuretics where the first group of effective antihypertensive drugs to be introduced in 1958 [15]. Since then, many medications have been developed. The treatment goals are to reduce the number of CVD-events and thereby to improve mortality. Different drug classes are currently available for the treatment of hypertension: 1. Diuretics (thiazides) [16]. Some of the most extensively used drugs in this class include bendroflumethiazide, hydrochlorothiazide and indapamide [17]; 2. Calcium channel blockers: Dihydropyridines such as amlodipine and nifedipine have specific impact on the vessels and induce a compensatory activation of baroreceptors [18]; 3. β-adrenoceptor antagonists: Metoprolol and atenolol with selectivity to β_1_-adrenoceptors and propranolol with no selectivity are some of the most common drugs of this class [19]; 4. Blockers of the renin-angiotensin-aldosterone system (RAAS): The group of angiotensin-converting-enzyme inhibitors (ACEi) includes drugs such as enalapril and captopril [20], angiotensin II receptor blockers (ARBs) include losartan, azilsartan and valsartan [21], and 5. Direct renin inhibitors comprise drugs like aliskiren [22].

## 3. Vitamin D

**Figure 1:**
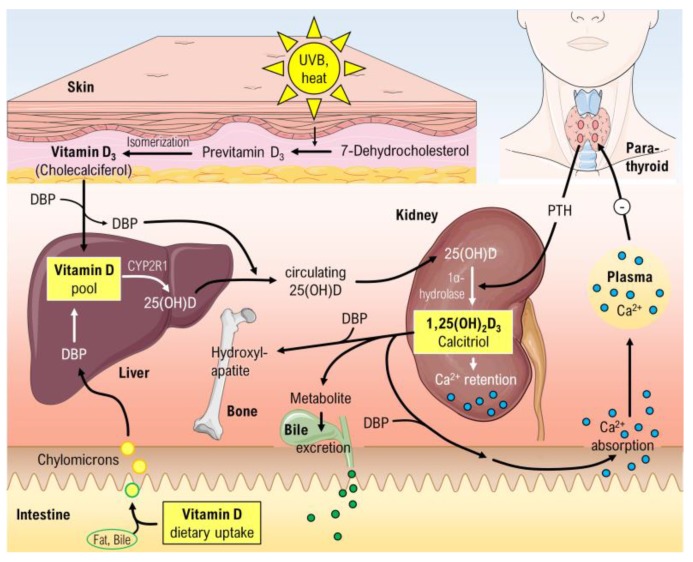
Overview of the vitamin D metabolism. 25(OH)D, Calcidiol; 1,25(OH)_2_D_3_, Calcitriol; CYP2R1, cytochrome P450 2R1; DBP, Vitamin D binding protein. Parts of the figure were drawn by using pictures from Servier Medical Art.

Vitamin D contains a steroid scaffold and possesses lipophilic properties. About 80–90% is endogenously synthetized and the remaining 10–20% come from nutritional intake. The metabolic pathways of vitamin D are shown in Figure 1. The inactive vitamin D is found in two distinct forms in the organism, cholecalciferol (D_3_) and ergocalciferol (D_2_) respectively. These inactive forms must undergo two hydroxylations to become the active form 1,25(OH)_2_D_3_. The endogenous synthesis of cholecalciferol is catalysed by sun-exposure of the skin with UVB radiation. In this process, 7-dehydrocholesterol is transformed to pre-vitamin D_3_, which isomerises to cholecalciferol. In the blood stream these two prohormones (D_3_ and D_2_) are bound to vitamin D binding protein (DBP). A hydroxylase enzyme in the liver, CYP2R1 (known as cytochrome P450 2R1), is responsible for converting D_2_ and D_3_ into 25(OH)D. The final activating step mainly takes place in the in proximal tubule cells, were 1α-hydroxylase (CYP27B1) converts 25(OH)D into 1,25(OH)_2_D_3_ named calcitriol. The last conversion step is highly regulated by feedback mechanisms [23].

### 3.1. The Vitamin D Receptor (VDR)

**Figure 2:**
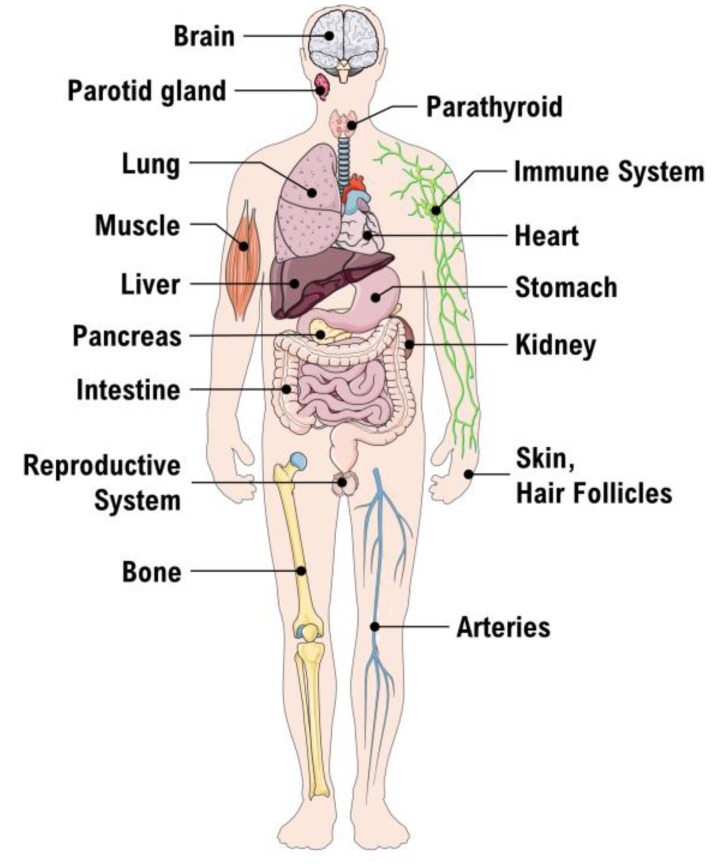
Tissue distribution of the Vitamin D receptor. Parts of the figure were drawn by using pictures from Servier Medical Art.

Brumbaugh et al. were the first to find evidence for the presence of a vitamin D receptor (VDR) [24]. This VDR is an intracellular receptor, able to bind 1,25(OH)_2_D_3_ and subsequently stimulate VDR to heterodimerize with retinoid X receptor (RXR) [23]. The VDR-RXR complex may translocate to the nucleus and bind to the regulatory site in the promoter region of DNA sequence elements. Thus, the gene expression of specific target genes will be regulated and facilitate synthesis of vitamin D-regulated proteins. The well-known biological effects of 1,25(OH)_2_D_3_ include absorption of Ca^2+^ from the small intestine, bone metabolism and calcium- and phosphorus homeostasis (Figure 1). The global function of the vitamin D system becomes clear, when we take a look at the VDR distribution in various tissues and cells of the human body (Figure 2) [25].

### 3.2. Vitamin D Status

The lipophilic nature of the vitamin D_3_ and the high binding affinity of 1,25(OH)_2_D_3_ to DBP in serum make these two substances difficult to use as markers. Thus, 25(OH)D concentration in plasma is considered the best measurement for vitamin D status [26].

Table 2 shows recommended doses of vitamin D intake and ideal serum 25(OH)D levels. However, there is an ongoing debate about the optimal levels of vitamin D. Intake reference values for vitamin D were developed by the Food and Nutrition Board (FNB) at the Institute of Medicine of The National Academies, USA. The Institute recommends 600 international units (IU) (15 µg) of vitamin D a day for adults ages 19 to 70. For adults age >70, the recommendation increases to 800 IU (20 µg) a day.

Adverse effects of vitamin D supplementation are rare. The initial signs of vitamin D intoxication are hypercalcemia, hypercalciuria and hyperphosphatemia [27]. In case of an overdose, hypercalcemia and/or hyperphosphatemia-associated symptoms can occur. Examples are weakness, fatigue, headache, appetite loss, dry mouth, nausea, vomiting, cardiovascular symptoms and others.

### 3.3. Suppression of Renin Production

In 2002, Li et al. designed an in vivo study of renin expression in VDR-null mice [28]. One group of wildtype mice (*n* = 9) were compared with a group of VDR-null mice (*n* = 8). The mice were given optimal growing conditions. After two months of age, they were put on a special diet for five weeks to normalize the calcium levels in plasma. Afterwards, the BP was measured under anaesthesia, renin-expression as well as the angiotensin (ANG) II activity were analysed. Interestingly, these analyses revealed a significantly higher (>20 mmHg) diastolic BP and SBP in VDR-null mice vs. wildtype mice. To obtain quantitative values of mRNA renin-expression, the Northern blot method was used. A significant 3.5-fold higher renin-expression and 2.5-fold higher serum level of ANG II in VDR-null mice vs. wildtype mice was seen (*p* < 0.001) [28].

To investigate the direct effects of active vitamin D on renin synthesis, another group of wildtype mice had five injections of 30 pmol 1,25(OH)_2_D_3_ in three following days. It turned out that the 1,25(OH)_2_D_3_ treatment gave a 50% reduction in renin-mRNA, when compared to the control group (*n* ≥ 3 in each group). Taken together, these findings imply the importance of vitamin D as an effective suppressor of the renin synthesis.

**Figure 3:**
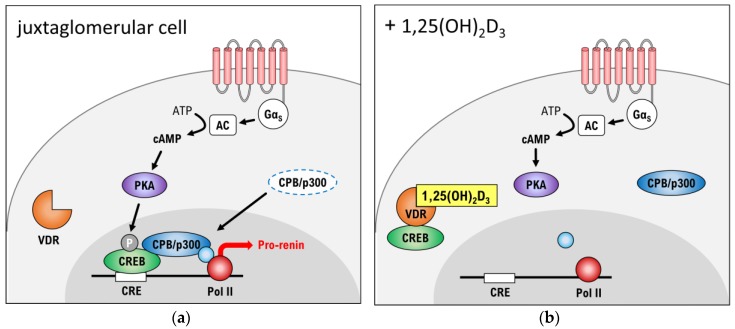
cAMP-PKA pathway. (**a**) Signalling in a juxtaglomerular cell in absence of 1,25(OH)_2_-vitamin D_3_; (**b**) Signalling in presence of 1,25(OH)_2_-vitamin D_3_. cAMP: cyclic adenosine monophosphate, CBP: CREB-binding protein, CRE: cAMP-dependent response element, CREB: cAMP response element-binding protein, Gα_S_: G_S_-protein alpha subunit, P: phosphate, PKA: protein kinase A, Pol II: RNA polymerase II, VDR: vitamin D receptor. The “+” stands for “JG in presence of”.

To elucidate the molecular pathways behind the downregulating effect of vitamin D on renin-transcription, Yuan et al. prepared an in vitro study [29]. In this study, specific juxtaglomerular As4.1 tumour cells from mice kidneys were analysed. In As4.1 cells the cyclic adenosine monophosphate (cAMP)/protein kinase A (PKA) pathway (shown in Figure 3) is deeply involved in the transcription of prorenin-mRNA. A Gα_S_-coupled protein activates adenylate cyclase (AC), which converts adenosine triphosphate (ATP) into cAMP. The elevated level of cAMP activates protein kinase A (PKA). The catalytic subunit of PKA then translocates to the nucleus, where it phosphorylates the cAMP response element-binding protein (CREB). Subsequently, CREB binds to its response element (CRE) in the promoter region of the *Ren1C* gene. The other co-activators CBP and p300 are recruited to form a CREB-CBP-CRE complex that promotes the gene transcription of pro-renin-mRNA.

Remarkably, 1,25(OH)_2_D_3_ liganded-VDR can interact directly with CREB to blunt its binding to CRE. It seems that these actions are carried out without the heterodimerization of liganded-VDR to RXR. Hence, it is believed that higher plasma levels of vitamin D can suppress the renin formation in juxtaglomerular cells [29]. However, another study indicates that decreased levels of renin in VDR-knockout mice will not cause a fall in BP [30]. Hence, it is important to state that more mechanisms must be implicated in a potential BP reducing effect of vitamin D.

## 4. Effects of Vitamin D on the Local Renin-Angiotensin System (RAS)

A local RAS is situated in several tissues, including heart, vessels, kidneys, lung, adrenal gland and nervous system [31]. The RAS acts in the control of cardiovascular, renal, and adrenal functions that regulate BP, body fluid and electrolyte status. It is known that the cardiac RAS is activated in cardiac pressure-overload and hypertensive rat models [32,33,34,35], and that the RAS plays an autocrine-paracrine role in the development of cardiac hypertrophy. VDR-deficient mice develop hypertension accompanied by an increase in heart weight. This finding reflects, at least in part, effects from activation of the systemic RAS [31].

Vitamin D is involved in cardiovascular protection, but only few studies examined the impact of the VDR in atherosclerosis. Macrophages express all components of the RAS, and are therefore involved in the process of atherosclerosis. It has been shown that macrophages in atherosclerotic lesions contain ANG II [36]. Another study showed that macrophage VDR signalling, in part by suppressing the local RAS, inhibits atherosclerosis in mice [37].

The antiatherosclerotic role of the VDR signalling in leukocytes/macrophages, and at least part of the antiatherosclerotic mechanism is to block the activation of the local RAS in macrophages and within the atherosclerotic lesion. Therefore, further studies to investigate the benefits of vitamin D and its analogues in atherosclerosis in both preclinical and clinical studies are desirable.

The intrarenal RAS is a key player for renal damage. Vitamin D deficiency activates the local RAS in the kidney, and thus it causes renal injury [38]. It is known that vitamin D is a negative RAS regulator by suppressing renin expression [28]. Moreover, vitamin D deficiency promotes the RAS [28], and a chronic RAS activation impacts lung function and induces a lung fibrosis [39]. Shi et al. showed in a recent paper that Vitamin D deficiency can induce profibrotic factors and activate the fibrotic cascade. These RAS-mediated effects are independent of an increased blood pressure [40]. In addition, there exists a local RAS in bone, which is involved in bone metabolism [41]. A recent study demonstrated that 1,25(OH)_2_D_3_ influences bone metabolism by downregulating the local bone RAS in a mice model with glucocorticoid-induced osteoporosis [42]. Vitamin D influences also the local pancreatic islet RAS and improves the islet beta cell secretory function [43]. A mice animal model with vitamin D receptor ablation revealed an activation of the islet RAS [43]. The application of calcitriol showed beneficial effects on the RAS activation under high-glucose conditions. In addition, it revealed a positive calcitriol effect on elevating the islet beta cell secretory response to glucose [43].

These animal studies show that vitamin D deficiency is a key player in different diseases. It influences the local RAS in various tissues. Vitamin D deficiency is an important health problem. Therefore, it is necessary to perform future studies to establish clinical guidelines for vitamin D supplementation required to achieve adequate vitamin D levels in people who are at risk for hypertension, atherosclerosis, cardiovascular disease, diabetes, pulmonary fibrosis, osteoporosis and others.

In particular, vitamin D deficiency might be linked to cardiovascular disease. There is a higher risk of high blood pressure (hypertension). However, more studies in this field are necessary. It is still too early to confirm and there is an ongoing discussion, whether a low vitamin D level causes hypertension or whether vitamin D supplementation will play a role in the treatment of hypertension. The role of vitamin D supplementation in the management or treatment of these diseases mentioned above must be studied in the future.

## 5. Vitamin D and Essential Hypertension

National Health and Nutrition Examination Survey (NHANES) is a survey providing information about health statistics in a representative sample of the U.S. population [44]. Based on the NHANES III data (survey period 1988–1994), Martins et al. examine the association between serum 25(OH)D-level and the prevalence of hypertension [45]. In this cross-sectional study, all individuals with available data and above 20 years of age (*n* = 15,088) are included. The study population is then divided into quartiles according to their serum 25(OH)-level. The 1st Quartile (25(OH)D < 21 ng/mL) shows a 20.46% prevalence of hypertension, while the 4th Quartile (25(OH)D ≥ 37 ng/mL) has a prevalence of 15.10%. Comparing the 1st to the 4th Quartile gives an odds ratio (OR) = 1.30 (95%CI: 1.13–1.48), adjusted for race, sex and age.

Scragg et al. use the same NHANES III data [46]. Here, individuals receiving anti-hypertensive medication are excluded and data is adjusted for physical activity, BMI, age, sex and race (*n* = 12,644). Both studies find a significant inverse correlation between BP and serum 25(OH)D level (*p* < 0.01). Ke et al. prepared a systematic review of the available observational studies in the period 2007–2014. It only includes published studies with healthy adults (*n* = 90,535) [2]. Comparing odds of hypertension from the top category to the bottom category of serum 25(OH)D level gives OR = 0.79 (95%CI: 0.73–0.87). This supports the hypothesis of an inverse relationship between hypertension and vitamin D status.

The strength of these studies is in particular due to the great sample size and it can be considered representative for the population. The disadvantages include the lack of temporal separation between data collection of exposure (vitamin D status) and outcome (hypertension). Hence, a causal relation can be difficult to establish even though a significant association is found. Furthermore, it can be hard to exclude the possibility of an inverse causality between exposure and outcome.

To meet the challenges of possible inverse causality between serum 25(OH)D level and hypertension, Kunutskor et al. reviewed all prospective studies in this field up until 2012 [47]. The included studies had to be representative for the population, with at least one year of follow-up and without prevalent hypertension at baseline (*n* = 283,537). Participants had serum 25(OH)D measured at baseline (*n* = 48,633) or vitamin D status assessed from dietary intake (*n* = 238,199). Hypertension during follow-up time was considered the endpoint.

Pooled relative risk (RR) in this review showed RR = 0.88 (95%CI: 0.81–0.97) i.e., a 12% reduction in risk of incident hypertension for every increase of 10 ng/mL in serum 25(OH)D level [47]. The group of participants with their vitamin D status based only upon the dietary intake had RR = 1.0 (95%CI: 0.95–1.05). Thus, no association between dietary vitamin D intake and risk of incident hypertension appeared. It is worth noting that dietary intake is a source of only 5–20% of vitamin D and its metabolites in plasma [48]. Therefore, it can be difficult to use the dietary intake as marker of vitamin D status, which can explain the lack of inverse correlation in this group.

To examine whether vitamin D supplementation is beneficial in hypertensive patients, the relevant studies are listed in Table 3. The included studies must meet the following criteria, to be discussed in review: participants should be adults (aged ≥18 years) with diagnosed vitamin D deficiency and/or arterial hypertension at baseline. Identified studies must be published or last updated in the time period 1 January 2012 until 27 November 2017. The intervention group must be administered cholecalciferol and participants may not suffer from preeclampsia. To minimize confounding, only RCT will be assessed.

In summary, animal studies had demonstrated that vitamin D deficiency is associated with high blood pressure by mechanisms revealing a direct effect on the renin-angiotensin-aldosterone axis. An altered vitamin D signalling in different animal models might be involved in cardiovascular diseases such as hypertension, cardiac hypertrophy, and atherosclerosis. Lower serum vitamin D was reported to be associated with higher BP levels in cross-sectional studies [46] and was associated inversely with blood pressure in a large sample representative of the US population [46]. This was supported by basic research. However, there are controversial results of basic research to randomized clinical trials.

A systematic review investigated whether vitamin D supplementation or its analogues may reduce blood pressure [57]. The trial search comprised a period from 1 January 1966, through 31 March 2014. 46 trials with 4541 participants were included in the trial-level meta-analysis [57]. The authors concluded that vitamin D supplementation is not effective as a drug for decreasing blood pressure and is not recommended as an antihypertensive drug [57].

It also has to be taken into account that genetic animal models may not completely reflect vitamin D deficiency of the human organism. Patients with vitamin D deficiency might suffer from chronic illnesses or have unknown cardiovascular risk factors. There might be dietary differences (diet with low vitamin D), differences in outdoor activities or sun-exposure. There is evidence suggesting that vitamin D application has little or no influence on BP, but to answer this point completely new trials are necessary. One example is the ongoing Vitamin D and Omega-3 Hypertension Trial (VITAL Hypertension) (NCT01653678) at Brigham and Women’s Hospital, Harvard Medical School, Boston, MA, USA. VITAL is an ongoing research study in 25,874 men and women across the U.S. The VITAL Hypertension study is being conducted among participants in VITAL and will examine whether vitamin D or omega-3 fatty acids are related to changes in BP and hypertension.

Another trial which is recruiting is the Vitamin D and Immune Mechanisms of Hypertension in Type 2 Diabetics (VDIM) trial (NCT03348280). This observational study will evaluate whether a particular type of circulating white blood cell, monocytes, from type 2 diabetics with high blood pressure and vitamin D deficiency vs. sufficiency will induce hormones that increase BP.

New international trials can inform about the best vitamin D supplementation dose, and to definitively evaluate the clinical utility of vitamin D therapy. Future studies should consider a design with a large sample size, control of medication, dietary salt and glucose intake, to avoid an activation of the RAAS. Large randomized and long-term trials focusing on patients with severe vitamin D deficiency and hypertension are needed before vitamin D can be recommended for prevention or treatment of hypertension.

The question of whether vitamin D deficiency can cause high BP unfortunately cannot be answered yet.

## 6. Discussion

The possible beneficial effects of Vitamin D supplementation on BP are the main focus of this review. As mentioned in the previous sections, epidemiological studies find some proof of an inverse correlation between serum vitamin D-status and prevalence of EH. However, the overview of interventional studies in Table 3 does not give a clear picture of vitamin D supplementation in relation to cardiovascular health. It is of interest whether vitamin D therapy in the intervention group (vitamin D deficiency at baseline) is able to raise serum 25(OH)D to the optimal levels (30–80 ng/mL) [58]. Common to the three RCTs is that they all show no effects from vitamin D supplementation in 24-h SBP [52,53,54]. These three studies share that the intervention groups at time endpoint are still insufficient in serum 25(OH)D levels. Common to Witham et al. trials is the high-dosage of oral administered cholecalciferol (100,000 IU) given at least two months apart [53,54]. This does not seem to restore the 25(OH)D levels, which can possibly explain the lack of effect on BP. In order to obtain positive effects of vitamin D therapy, there is something that suggests the dose interval to be short (e.g., daily or weekly administration).

McGreevy et al. trial participants were given intramuscular injections of 100,000 IU cholecalciferol, which did not restore serum 25(OH)D levels [52]. One possible explanation could be a diminished bioavailability. However, the primary outcome ‘arterial stiffness’ was improved after intervention. This could probably be carried out by an unknown VDR-RXR-protective mechanism in the endothelial wall, independently of renin secretion and the RAAS. Cipriani et al. conclude high-dosage of p.o. administered cholecalciferol to be more efficiently in raising serum 25(OH)D levels, when compared to equivalent intramuscular injections [59]. Hence, future study designs should rather use oral administration than intramuscular injections in the intervention groups.

The DAYLIGHT trial [50] and Styrian trial [49] have the biggest sample size (*n* = 534 and *n* = 200) among the RCTs for this review. These trials find no significant changes in measured BP outcomes after a daily administration of vitamin D in the study period.

Larsen et al. found in the overall group no significant BP beneficial effects of vitamin D. Nevertheless, subgroup analysis revealed significant 24-h BP reductions in a subgroup with vitamin D-insufficiency at baseline [51]. One might suggest that there could be a certain threshold in serum 25(OH)D to achieve cardiovascular benefits. Interestingly, Mozaffari-Khosravi et al. observed a remarkably increment in serum 25(OH)D levels and significant MAP reductions at same time [56]. On average, serum levels of 25(OH)D were raised to 51.7 ng/mL in intervention group, which could give support to the idea of a dose-response relation. Hence, study designs with lack of satisfying restoration of serum 25(OH)D levels will fail to improve BP.

Chen et al. investigate the administration of vitamin D as ‘add-on’ to a nifedipine treatment [55]. This combined therapy seems to markedly reduce systolic and diastolic BP—but further studies are needed to elucidate the effects of cholecalciferol and antihypertensive drugs combined.

## 7. Conclusions and Outlook

Essential hypertension is a condition, in which an imbalance between vasoconstriction and vasodilation occurs. More factors, both epigenetic and environmental, are related to the development of EH. Many epidemiological studies find that vitamin D deficiency can be associated to prevalent hypertension. Furthermore, experimental studies in mice can explain a possible mechanism behind a CVD-protective effect of vitamin D supplementation. Hence, vitamin D injections were shown to inhibit the renin synthesis. This was mediated by the liganded-VDR, which could interact with specific transcription factors to reduce renin transcription. Cholecalciferol can thus be a potentially cost-effective antihypertensive drug.

However, clinical trials in this area show conflicting results—which in part can be attributed to suboptimal study designs. Hence, further studies that efficiently restore serum vitamin D status, are desirable. 

Future aspects in this research field include investigating if vitamin D analogues with higher selectivity to VDR could have BP lowering effects. The potential synergistic effect of cholecalciferol and antihypertensive drugs also needs to be further investigated.

## Figures and Tables

**Table 1:** Classification of hypertension.

Classification	SBP (mmHg)	Header	DBP (mmHg)
Normal	<120	and	<80
Elevated	120–129	and	<80
Hypertension, Stage 1	130–139	or	80–89
Hypertension, Stage 2	≥140	or	≥90

**Table 2:** Vitamin D recommendations.

Recommendation	Children and Adolescents	Adults
The optimal concentration of 25(OH)D in plasma	20–60 ng/mL ≈ 50–150 nM	30–80 ng/mL ≈ 75–200 nM
Supplementations: recommended dose when severe deficiency	up to 5000 IU/day = 125 µg/d	up to 7000 IU/day ≈ 175 µg/d

**Table 3:** Overview of recent clinical trials with vitamin D intervention in hypertensive patients.

Title	Design	Objective	Conclusions
The Styrian Vitamin D Hypertension Trial: effects of vitamin D on blood pressure and cardiovascular risk factors. [49] NCT02136771	Randomized, Double-blind, Placebo-controlled, *n* = 200	To assess the effects on 24-h systolic BP and diastolic BP of vitamin D_3_ supplementation of 2800 IU/day for 8 weeks in vitamin D deficient individuals.	There was no significant effect on systolic and diastolic BP after treatment with vitamin D supplementation.
Vitamin D therapy in individuals with prehypertension or hypertension: the DAYLIGHT trial. [50] NCT01240512	Randomized, Double-blind, Parallel assignment, Multi-center, *n* = 534	To compare the BP-lowering effects of high-dose (4000 IU/day) vs. low-dose (400 IU/day) of cholecalciferol for 6 months in vitamin D deficient individuals. The participants were prehypertensive or hypertensive at baseline and had not been taking antihypertensive drugs.	No significant changes in BP-measures was observed in the two groups. Nevertheless, a non-significant (*p* = 0.71) decrease in 24-h SBP was observed −0.8 mmHg and −1.6 mmHg in the two groups, respectively.
Effect of Vitamin D replacement During Winter Months in Patients With Hypertension. [51] NCT01166165	Randomized, Double-blind, Placebo-controlled, *n* = 130	To investigate therapeutic effects of 3000 IU/day cholecalciferol for 20 weeks in hypertensive patients.	In the overall group, no significant reductions in 24-h-BP, when compared to placebo. A subgroup analysis containing only deficient plasma−25(OH)D (<32 ng/mL) individuals at baseline, showed a significant reduction in 24-h systolic/diastolic BP −4/−3 mmHg.
The effect of vitamin D supplementation on arterial stiffness in an elderly community-based population. [52] EUDRA number: 2010–024417–31	Randomized, Double-blind, Parallel assignment, *n* = 119	To compare the effects of 50,000 IU vs. 100,000 IU single-dose intramuscular injection of cholecalciferol in a group of elderly people.	8 weeks after treatment the group receiving high-dose of cholecalciferol had a significant improvement in arterial stiffness compared to the low-dose group. At the same time, systolic BP seemed to elevate in high-dose group.
Vitamin D therapy to reduce blood pressure and left ventricular hypertrophy in resistant hypertension. [53] EUDRA number: 2008-002681-63	Randomized, Double-blind, Placebo-controlled, *n* = 68	To assess effects of high-dose cholecalciferol supplementation (100,000 IU every 2^nd^ month for 6 months) in patients treated with ≥3 antihypertensive drugs.	The study showed no improvements in systolic or diastolic BP after 6 months of treatment.
Cholecalciferol treatment to reduce blood pressure in older patients with isolated systolic hypertension: the VitDISH randomized controlled trial. [54] ISRCTN92186858	Randomized, Double-blind, Placebo-controlled, *n* = 159	To explore the effects on BP of high-dose cholecalciferol treatment (100,000 IU every 3rd month for 1 year) in elderly patients with isolated systolic hypertension	Treatment did not reduce BP or improve other secondary cardiovascular outcomes.
Vitamin D and nifedipine in the treatment of Chinese patients with grades I-II essential hypertension: a randomized placebo-controlled trial. [55] ChiCTR-ONC-13003840	Randomized, Double-blind, Placebo-controlled, *n* = 126	To assess the effects of cholecalciferol (2000 IU/day for 6 months) as ‘add on’ to nifedipine in essential hypertensive patients.	Cholecalciferol as ‘add on’ gave a significant systolic/diastolic BP reduction (−6.2/−4.2 mmHg). In subgroup analysis of vitamin D insufficient (at baseline) patients, showed −7.1/−5.7 mmHg BP reduction (*p* = 0.001).
The effect of vitamin D supplementation on blood pressure in patients with elevated blood pressure and vitamin D deficiency: a randomized, double-blind, placebo-controlled trial. [56]	Randomized, Double-blind, Placebo-controlled, *n* = 42	To assess BP lowering effect of cholecalciferol 50,000 IU/week for 8 weeks in hypertensive, vitamin D deficient patients.	In the vitamin D deficient group (VDG) 92.7% of individuals recovered from insufficiency. Middle arterial pressure (MAP) decreased in average −3.7 mmHg in VDG and increased 0.9 mmHg in place-controls (*p* < 0.001).

## Abbreviations

1,25(OH)_2_D_3_	Calcitriol
25(OH)D	Calcidiol
95%-CI	95% confidence interval
AC	Adenylate cyclase
ACE	Angiotensin-converting enzyme
ACEi	Angiotensin-converting enzyme inhibitor
ANG	Angiotensin
ARB	Angiotensin II receptor blocker
AT_1_/AT_2_	Angiotensin receptor type 1 or 2
BMI	Body mass index
BP	Blood pressure
cAMP	cyclic adenosine monophosphate
CBP	CREB-binding protein
CO	Cardiac output
CRE	cAMP response element
CREB	cAMP-dependent response element binding protein
CVD	Cardiovascular disease
CYP	Cytochrome P450
DBP	Vitamin D binding protein
EH	Essential hypertension
HR	Heart rate
IU	International units
MAP	Mean arterial pressure
mRNA	messenger ribonucleic acid
NHANES	National Health and Nutrition Examination Survey
OR	Odds-ratio
PKA	Protein kinase A
RAAS	Renin-angiotensin-aldosterone system
RCT	Randomized controlled trial
RR	Relative risk
RXR	Retinoid X receptor
SBP	Systolic blood pressure
TPR	Total periphery resistance
VDR	Vitamin D receptor
WHO	World Health Organization
